# Gene Expression of Extracellular Matrix Proteins, MMPs, and TIMPs in Post-Operative Tissues of Chronic Rhinosinusitis Patients

**DOI:** 10.3390/cells14090654

**Published:** 2025-04-29

**Authors:** Zygimantas Vaitkus, Astra Vitkauskiene, Liutauras Labanauskas, Justinas Vaitkus, Povilas Lozovskis, Saulius Vaitkus, Ieva Janulaityte

**Affiliations:** 1 Department of Ear, Nose and Throat, Faculty of Medicine, Academy of Medicine at Lithuanian University of Health Sciences, LT-44307 Kaunas, Lithuania; zygimantas.vaitkus@lsmu.lt (Z.V.); justinas.vaitkus@lsmu.lt (J.V.); saulius.vaitkus@lsmu.lt (S.V.); 2 Department of Laboratory Medicine, Faculty of Medicine, Academy of Medicine at Lithuanian University of Health Sciences, LT-44307 Kaunas, Lithuania; astra.vitkauskiene@lsmu.lt (A.V.); povilas.lozovskis@lsmu.lt (P.L.); 3 Department of Pediatrics, Faculty of Medicine, Academy of Medicine at Lithuanian University of Health Sciences, LT-44307 Kaunas, Lithuania; liutauras.labanauskas@lsmu.lt

**Keywords:** extracellular matrix, collagen, fibronectin, MMP, TIMP, chronic rhinosinusitis, nose polyposis, gene expression

## Abstract

Chronic rhinosinusitis (CRS) is a persistent inflammatory condition of the sinus mucosa characterized by significant tissue remodeling. This study aimed to evaluate the gene expression of extracellular matrix (ECM) proteins, matrix metalloproteinases (MMPs), and tissue inhibitors of metalloproteinases (TIMPs) in post-operative tissues of CRS patients. A total of 30 patients diagnosed with CRS, divided into CRSwNP (with nasal polyps) and CRSsNP (without nasal polyps) groups, were compared with a control group of 10 individuals undergoing nasal surgeries for non-CRS conditions. Gene expression analysis was conducted using quantitative real-time PCR, and plasma cytokine levels were measured via ELISA. Results indicated significantly higher expression of *collagen I*, *collagen III*, *fibronectin*, *vimentin*, *periostin*, and *tenascin C* in CRS tissues, especially in CRSsNP patients. Conversely, *elastin* expression was markedly lower. *MMP-2*, *MMP-9*, *TIMP-1*, and *TIMP-2* expression was significantly altered, with CRSsNP showing lower levels compared to CRSwNP and controls. *TGF-β1* expression was elevated in both CRS groups, particularly in CRSsNP, highlighting its role in fibrosis and ECM remodeling. Additionally, increased plasma concentrations of TSLP and TGF-*β*1 suggest epithelial activation and immune dysregulation in CRS. These findings underscore distinct remodeling profiles in CRS endotypes, emphasizing the need for targeted therapeutic strategies based on molecular phenotyping. Understanding ECM dysregulation and inflammatory pathways in CRS may lead to improved, individualized treatment approaches.

## 1. Introduction

Chronic rhinosinusitis (CRS) is a chronic inflammatory disease of the sinus or nasal passages for over 12 continuous weeks. Unlike acute rhinosinusitis, primarily caused by infections, CRS is an inflammatory disease resulting from multiple contributing factors [1]. CRS is prevalent among all age groups and is the fifth most common reason for an antibiotic prescription [2,3,4]. Patients present with cardinal symptoms of sinusitis, which are purulent nasal discharge, facial or dental pain, and nasal obstruction. CRS may present as chronic sinusitis without nasal polyposis (CRSsNP), chronic sinusitis with nasal polyposis (CRSwNP), and allergic fungal rhinosinusitis [5,6]. In recent years, CRS has been further classified into type 1 (T-helper (Th)1 inflammation), type 2 (Th2 inflammation), and type 17 (Th17 inflammation) inflammation, focusing on the immune response characteristics of CRS [7]. In recent decades, CRSsNP has been recognized for causing a neutrophil-dominant Th1/Th17 inflammatory response, while CRSwNP leads to an eosinophil-dominant Th2 inflammatory response [8,9,10,11,12].

Tissue remodeling is the process by which the structure and function of damaged areas in all body organs are restored [13]. Tissue remodeling is a dynamic phenomenon that can result in temporary or permanent changes. Ongoing inflammation caused by a dysregulated immune response can lead to the pathological accumulation of extracellular matrix (ECM). ECM dysregulation in CRS is suggested by genome-wide association studies identifying ECM genes as top candidates [14]. This issue was first observed in the lower respiratory tract, commonly associated with chronic inflammatory diseases such as asthma and chronic obstructive pulmonary disease [15,16,17]. CRS induces mucosal tissue remodeling, including ECM accumulation, fibrin deposition, edema, immune cell infiltration, and neoangiogenesis [18]. Biopsy samples usually reveal a thickened basement membrane, goblet cell hyperplasia, atypical gland architecture, and infiltration with monocytes, neutrophils, and eosinophils. The differentiation of nasal fibroblasts into myofibroblasts, as well as ECM production and tissue remodeling, is well established by TGF-*β*, a potent cytokine that induces fibrosis that is widely secreted by eosinophils and fibroblasts [18,19,20,21]. As few studies currently comprehensively assess potential changes in ECM protein expression and production, our study aimed to evaluate protein targets that may contribute to promoting inflammation in CRS.

There is no consensus on an approach to the management of chronic sinusitis. The treatment should focus on modulating triggers, reducing inflammation, and eradicating the infection [2,22]. Recent evidence suggests that upper respiratory tract remodeling characteristics occur depending on the endogenous type. Understanding this may offer great potential for the customized treatment of CRS [23]. This study sought to examine the gene expression of ECM proteins, MMPs, TIMPs, and certain cytokines in post-operative tissues from CRSwNP and CRSsNP patients, aiming to uncover remodeling differences that could enhance the understanding of CRS remodeling.

## 2. Materials and Methods

The research protocol was approved on 3 May 2023 by the Kaunas Regional Biomedical Research Ethics Committee of the Lithuanian University of Health Sciences with permission no. BE-2-10. The research study was registered in the ISRCTN (International Standard Randomised Controlled Trial Number) database registry with the identifier ISRCTN36938545 https://doi.org/10.1186/ISRCTN36938545 (accessed on 23 April 2025). This study is an experimental study based on prospectively collected post-operative tissue samples.

Patients were selected in 2023–2024 at the Department of Otorhinolaryngology of the Lithuanian University of Health Sciences Hospital Kauno Klinikos. This study included only non-smokers at least 18 years of age who signed the informed consent form. Criteria for excluding patients from this study: younger than 18, pregnant women, those with autoimmune or chronic lung diseases, and previous or current leukemias. A total of 30 individuals who had CRS were recruited for this study and were confirmed with a diagnosis based on endoscopic and CT scan results, confirming the diagnosis based on the European position on rhinosinusitis and nasal polyps 2020 set criteria [24]. These patients are classified into two groups: CRSwNP and CRSsNP, as the treatment strategy typically relies on the presence of polyposis. The control group consisted of 10 individuals who underwent surgery on other diseases or pathologies, including nasal septum deviation, dacryoadenitis, and turbinate hypertrophy.

### 2.1. Sample Collection and Preparation

The post-operative tissues of the studied patients were collected in Eppendorf-type 1.5 mL RNases, DNases, and pyrogen-free tubes. The post-operative tissues are divided into several parts; for gene expression analysis, pieces no larger than 30 mg were used and homogenized using a scalpel cut and mechanical crushing in a Petri dish. After cutting and crushing the RLT buffer from Qiagen RNeasy^®^ Mini Kit (Qiagen, Redwood City, CA, USA) for cell lysing was used, further procedures were performed according to the manufacturer’s instructions. For the evaluation of IL-6, TSLP, and TGF-*β*1 concentrations in plasma samples, the plasma was collected and stored until analysis, following the manufacturer’s instructions.

The blood of the studied patients was collected in tubes with K2EDTA (ethylenediaminetetraacetate) (BD Bioscience, San Jose, CA, USA). A complete blood count test was performed using the Mindray BC 6800 Plus automatic hematologic analyzer (Shenzhen Mindray Bio-Medical Electronics, Shenzhen, China).

The blood of the studied patients was collected in serum tubes with a clot activator (BD Bioscience, San Jose, CA, USA). The concentration of IgE in the blood was measured using AIA-2000 (TOSOH BIOSCIENCE, South San Francisco, CA, USA).

### 2.2. RNA Isolation and Quantitative Real-Time PCR Analysis

RNA was isolated using the Qiagen RNeasy^®^ Mini Kit (Qiagen, CA, USA). RNeasy technology simplifies the isolation of total RNA, ensuring the proper quality and quantity of RNA. The samples of post-operational material was used for gene expression analysis. For this study, all tissue samples were obtained from the mucosal surface of the middle and/or inferior turbinates during endoscopic sinus surgery. Nasal polyps were excluded from the collection to ensure the analysis of non-polypoid sinonasal mucosa. Identical sites were used for histological evaluation to maintain consistency across samples. The samples are first lysed and then homogenized. Ethanol is added to the lysate to create ideal conditions for RNA binding. Then, the lysate is transferred to the silica membrane of the RNeasy column, where RNA binds to the silica dioxide membrane, and all other components are effectively washed away according to the manufacturer’s protocol. Pure, concentrated RNA is eluted in deionized, RNase, DNase and pyrogen free water. The purity and concentration of the separated RNA are measured using the EpochTM microplate spectrophotometer (Epoch, BioTek Instruments, Winooski, VT, USA).

Quantitative real-time polymerase chain reaction (qRT-PCR) was performed using the 7500 Fast Real-Time PCR System with a PowerSYBR^®^Green RNA-to-CT™ 1-Step Kit (Applied Biosystems, Foster City, CA, USA) according to the manufacturer’s protocol. The primers used for gene expression analysis are listed in Table 1.

Gene expression levels were quantified using the ΔΔCt method, where ΔCt values (target gene normalized to 18S rRNA) of the experimental group were compared to those of the control group. Negative ΔΔCt values indicate upregulation, whereas positive values indicate downregulation of the target gene relative to the control condition.

### 2.3. Evaluation of IL-6, TSLP, and TGF-β1 Concentration in Plasma

All study groups used plasma samples to evaluate IL-6, TSLP, and TGF-*β*1 concentration. The preparation of platelet-free plasma included the centrifugation of K2EDTA anticoagulant tubes. The separated blood plasma is transferred to Eppendorf-type tubes and centrifuged repeatedly for 15 min at 10,000× *g* at 4 °C, as stated in the ELISA protocol. Then, plasma was collected into new Eppendorf-type tubes, leaving a small amount of plasma and sediment formed on the bottom. After double centrifugation, the collected plasma is frozen at −20 °C until the ELISA test is performed.

For ELISA following reagents were used: Human IL-6 ELISA kit (Catalog No: BSKH1007 Bioss Inc., Woburn, MA, USA); Human TSLP ELISA kit (Catalog No: BSKH1031 Bioss Inc., Woburn, MA, USA); Human TGF-*β*1 ELISA kit (Catalog No: BSKH60051 Bioss Inc., Woburn, MA, USA). The optical density was determined using a microplate reader (Epoch, BioTek Instruments, Winooski, VT, USA). Each test sample was performed in two repetitions.

### 2.4. Statistical Analysis of Data

The data were analyzed using GraphPad Prism 10 for Windows (GraphPad Software, Inc., San Diego, California, USA). A test for normality was performed using the Shapiro–Wilk test. Given the small sample size, nonparametric statistical methods were employed. The Mann–Whitney *U* test identified statistically significant differences between the test groups. A single-sample Wilcoxon test was also used to evaluate differences in gene expression between the CRS groups and the control. For correlation, the Spearman r test and nonlinear regression were used to find possible associations between clinical and experimental data. The results were presented as the mean ± standard deviation. The findings were considered statistically significant when *p* < 0.05.

## 3. Results

### 3.1. Characteristics of the Study Population

Forty non-smoking subjects (25 males and 15 females) were included in the biomedical study: 15 subjects with CRSsNP, 15 subjects with CRSwNP, and 10 subjects as the control group that was treated and operated on in the Department of Otorhinolaryngology of Lithuanian University of Health Sciences Hospital Kauno klinikos for other cases than CRS. It is important to note that none of the CRS patients were treated previously. In the control group, six subjects had nasal septum deviation, three had dacryoadenitis, and one had turbinate hypertrophy. Table 2 presents the clinical and demographic data of the study subjects. In complete blood count, significant differences were found in the absolute count of basophils in CRSsNP and the control group; the significantly higher numbers were found compared to CRSwNP, accordingly 0.027 ± 0.015 vs. 0.018 ± 0.014 × 10^9^, *p* = 0.0387; 0.031 ± 0.020 vs. 0.018 ± 0.014 × 10^9^, *p* = 0.0406. Furthermore, the eosinophils percentage was significantly higher in the CRSwNP group compared to the control group, 2.247 ± 1.212 vs. 1.400 ± 1.173 %, *p* = 0.0413. Due to difficulties in collecting anamnesis of allergic disease, IgE concentrations had no differences between groups and, at the same time, had extremely high values in both CRS groups, thus suggesting that allergic processes are possible in both CRS groups. Sinonasal outcome test questionnaire score was significantly higher in CRSsNP and CRSwNP groups compared to control, accordingly 45.53 ± 18.10 vs. 14.80 ± 11.22, *p* = 0.0003; 47.73 ± 23.75 vs. 14.80 ± 11.22, *p* < 0.0001.

Cytology samples from nasal discharge and biopsies for histological examination were collected from all patients across both CRS groups during surgery. In the control group, only eight nasal discharge samples were collected, with no biopsies taken for further histological analysis. The samples underwent examination according to the routine diagnostic and treatment protocols established by the Department of Pathology at the Lithuanian University of Health Sciences Hospital Kauno klinikos. The results indicated that the nasal eosinophilia group presented more cases of CRSwNP than the other groups, as shown in Table 3; however, no significant differences were identified. Lymphocytes, plasmocytes, and macrophages were exclusively observed in the CRSsNP group. Regarding histological findings, hyperplasia and neutrophilia were more prevalent in cases of CRSsNP compared to CRSwNP, whereas vascularization and eosinophilia were more common in CRSwNP than in CRSsNP.

### 3.2. Gene Expression of Extracellular Matrix Proteins

**Figure 1:**
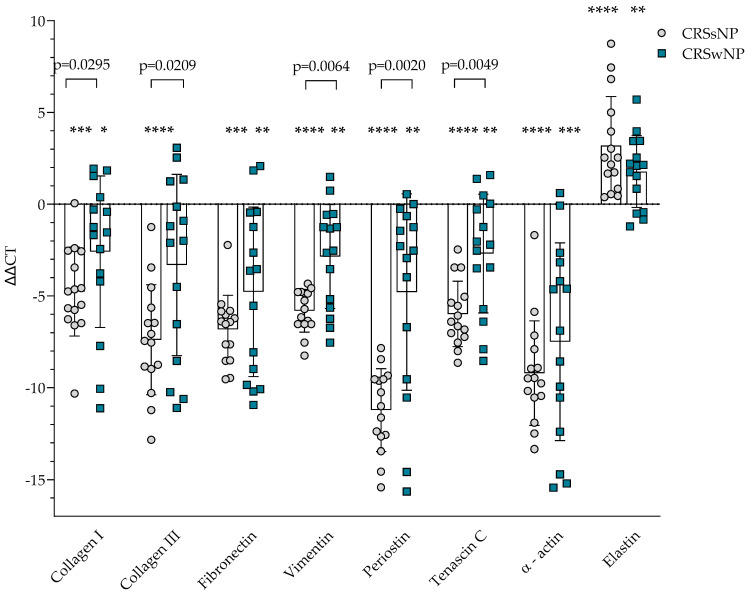
Gene expression of extracellular matrix protein in post-operative material from patients with CRSsNP and CRSwNP. Data presented as the mean ± SD, ΔΔCT over control subjects. CRSsNP—chronic rhinosinusitis without nasal polyposis; CRSwNP—chronic rhinosinusitis with nasal polyposis. CRSsNP n = 15; CRSwNP n = 15; control group n = 10. * *p* < 0.05 compared with control group; ** *p* < 0.01 compared with control group; *** *p* < 0.001 compared with control group; **** *p* < 0.0001 compared with control group. Lines connect comparison groups with a *p*-value denoting the significant difference and pair-wise comparisons. Statistical analysis between investigated groups—two-sided Mann–Whitney *U* test (independent data); Wilcoxon signed-rank test used for gene expression analysis against the control group.

The gene expression of ECM proteins was evaluated in the post-operational material from CRSsNP, CRSwNP, and the control group. Gene expression of *collagen I*, *collagen III*, *fibronectin*, *vimentin*, *periostin*, *tenascin C*, and *α-actin* was significantly higher in CRSsNP and CRSwNP compared to the control group, *p* < 0.05. In comparison, gene expression of *elastin* was significantly lower in CRSsNP and CRSwNP groups compared to the control group, *p* < 0.05 (Figure 1). Gene expression of *collagen I*, *collagen III*, *vimentin*, *periostin*, *tenascin C* was significantly higher in CRSsNP compared to CRSwNP group, accordingly *collagen I* −4.764 ± 2.422 vs. −2.588 ± 4.129 ΔΔCT over the control group, *p* = 0.0295; *collagen III* −7.388 ± 2.994 vs. −3.313 ± 4.942 ΔΔCT over the control group, *p* = 0.0209; *vimentin* −5.806 ± 1.162 vs. −2.852 ± 2.828 ΔΔCT over the control group, *p* = 0.0064; *periostin* −11.222 ± 2.252 vs. −4.788 ± 5.533 ΔΔCT over the control group, *p* = 0.0020; *tenascin C* −5.982 ± 1.777 vs. −2.690 ± 3.234 ΔΔCT over control group, *p* = 0.0049.

### 3.3. Gene Expression of MMP and TIMP

**Figure 2:**
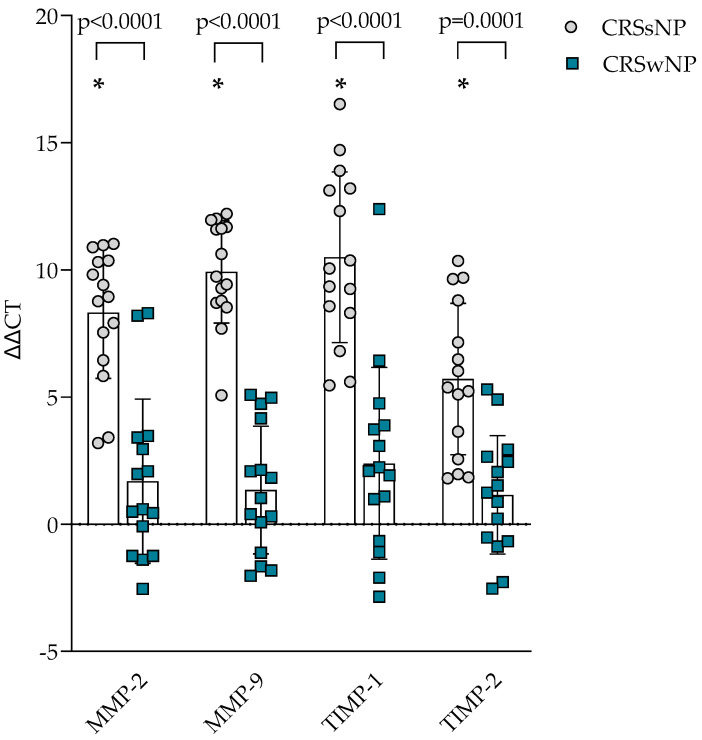
Gene expression of MMP and TIMP in post-operative material from patients with CRSsNP and CRSwNP. Data presented as the mean ± SD, ΔΔCT over control subjects. CRSsNP—chronic rhinosinusitis without nasal polyposis; CRSwNP—chronic rhinosinusitis with nasal polyposis; MMP—matrix metalloproteinases; TIMP—tissue inhibitor of metalloproteinases. CRSsNP n = 15; CRSwNP n = 15; control group n = 10. * *p* < 0.0001 compared with control group. Lines connect comparison groups with a *p*-value denoting the significant difference and pair-wise comparisons. Statistical analysis between investigated groups—two-sided Mann–Whitney *U* test (independent data); Wilcoxon signed-rank test used for gene expression analysis against the control group.

The gene expression of ECM homeostasis regulating MMPs and TIMPs was evaluated in the post-operational material from CRSsNP, CRSwNP, and the control group and presented in Figure 2. It was found that *MMP-2*, *MMP-9*, *TIMP-1*, and *TIMP-2* gene expression in post-operational material was significantly lower in the CRSsNP group compared to the control group, *p* < 0.0001, and CRSwNP group, accordingly *MMP-2* 8.321 ± 2.591 vs. 1.692 ± 3.230 ΔΔCT over the control group, *p* < 0.0001; *MMP-9* 9.928 ± 2.017 vs. 1.344 ± 2.509 ΔΔCT over the control group, *p* < 0.0001; *TIMP-1* 10.50 ± 3.357 vs. 2.392 ± 3.775 ΔΔCT over the control group, *p* < 0.0001; *TIMP-2* 5.710 ± 2.980 vs. 1.152 ± 2.327 ΔΔCT over the control group, *p* < 0.0001.

### 3.4. Gene Expression of TGF-β1

**Figure 3:**
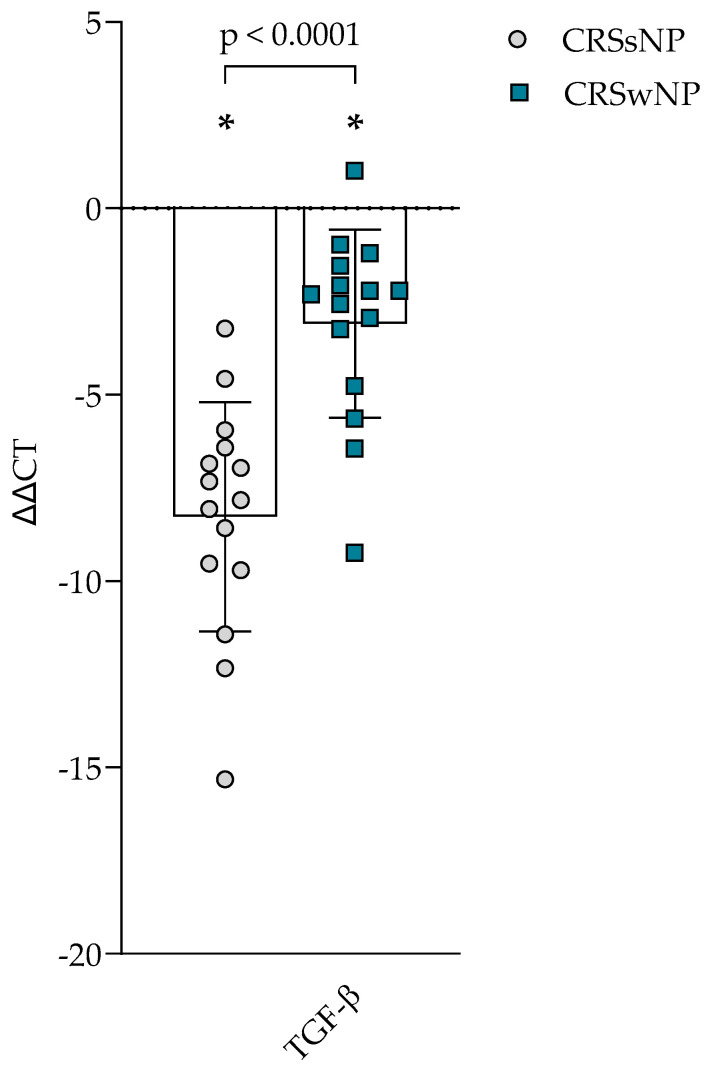
Gene expression of TGF-*β* in post-operative material from patients with CRSsNP and CRSwNP. Data presented as the mean ± SD, ΔΔCT over control subjects. CRSsNP—chronic rhinosinusitis without nasal polyposis; CRSwNP—chronic rhinosinusitis with nasal polyposis; TGF-*β*—transforming growth factor *β*. CRSsNP n = 15; CRSwNP n = 15; control group n = 10. * *p* < 0.0001 compared with control group. Lines connect comparison groups with a *p*-value denoting the significant difference and pair-wise comparisons. Statistical analysis between investigated groups—two-sided Mann–Whitney U test (independent data); Wilcoxon signed-rank test used for gene expression analysis against the control group.

The gene expression of *TGF-β1* was evaluated in the post-operational material from CRSsNP, CRSwNP, and the control group and presented in Figure 3. Gene expression in CRSsNP and CRSwNP was significantly higher than in the control group, *p* < 0.0001. When comparing CRS groups, it was found that CRSsNP had significantly higher *TGF-β1* gene expression in post-operational material than CRSwNP, accordingly −8.276 ± 3.078 vs. −3.091 ± 2.524 ΔΔCT over the control group, *p* < 0.0001.

### 3.5. Concentration of IL-6, TSLP and TGF-β1 in Plasma

**Figure 4:**
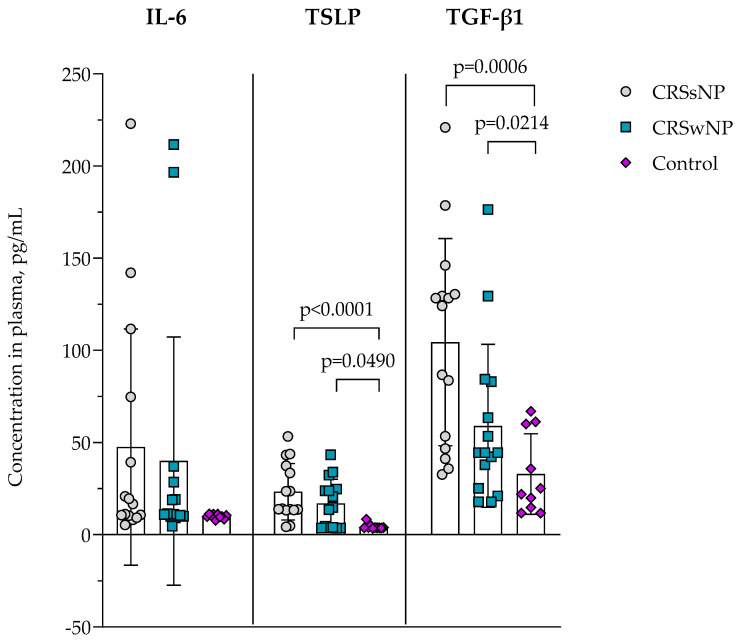
The concentration of IL-6, TSLP, and TGF-*β* in plasma. Data presented as the mean ± SD. CRSsNP—chronic rhinosinusitis without nasal polyposis; CRSwNP—chronic rhinosinusitis with nasal polyposis; IL-6—interleukin 6; TGF-*β*—transforming growth factor *β*; TSLP—thymic stromal lymphopoietin. CRSsNP n = 15; CRSwNP n = 15; control group n = 10. Lines connect comparison groups with a *p*-value denoting the significant difference and pair-wise comparisons. Statistical analysis between investigated groups—two-sided Mann–Whitney *U* test (independent data).

The concentration of IL-6, TSLP, and TGF-*β*1 in the plasma of CRSsNP, CRSwNP, and the control group was evaluated and presented in Figure 4. In IL-6, no differences between groups were found. TSLP concentration was found to be higher in the CRSsNP group compared to the control group, 23.280 ± 15.310 vs. 4.293 ± 1.480 pg/mL, *p* < 0.0001; and in CRSwNP was significantly higher compared to the control group, 16.920 ± 13.200 vs. 4.293 ± 1.480 pg/mL, *p* = 0.0490. TGF-*β*1 concentration in plasma was found to be higher in the CRSsNP group than in the control group, 104.400 ± 56.190 vs. 32.890 ± 21.830 pg/mL, *p* = 0.0006, and in CRSwNP was significantly higher compared to the control group, 59.020 ± 44.190 vs. 32.890 ± 21.830 pg/mL, *p* = 0.0214.

### 3.6. Integration of Clinical and Molecular Data

**Figure 5:**
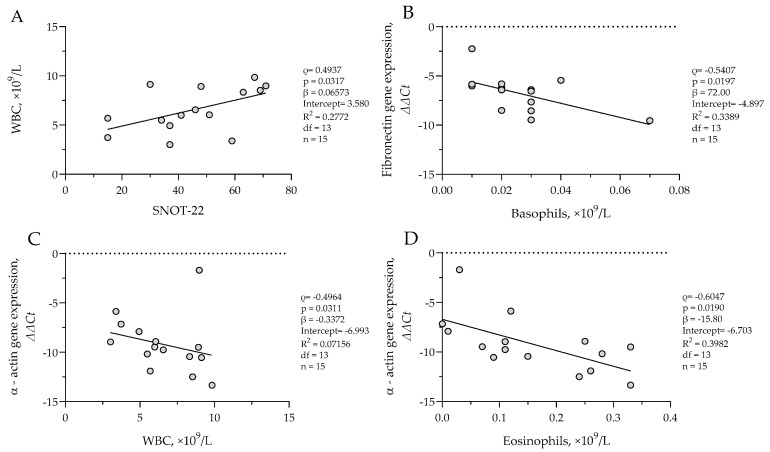
(**A**) Correlation between WBC count and SNOT-22 score in CRSsNP group; (**B**) correlation between *fibronectin* gene expression and basophil count in CRSsNP group; (**C**) correlation between *α-actin* gene expression and WBC count in CRSsNP group; (**D**) correlation between *α-actin* gene expression and eosinophil count in CRSsNP group. SNOT-22—sinonasal outcome test; WBC—white blood cells. Gene expression data are presented as ΔΔCt, with lower ΔΔCt values indicating higher gene expression. CRSsNP n = 15. Statistical analysis—Spearman r test and nonlinear regression.

In the CRSsNP group, a positive correlation was observed between SNOT-22 scores and WBC count (*ρ* = 0.4937, *p* = 0.0317; *β* = 0.06573, *R*^2^ = 0.2772), suggesting that greater symptom burden was associated with higher systemic inflammatory activity. Basophil counts negatively correlated with *fibronectin* ΔΔCt values (*ρ* = −0.5407, *p* = 0.0197; *β* = 72.00, *R*^2^ = 0.3389), while both WBC count (*ρ* = −0.4964, *p* = 0.0311; *β* = −0.3372, *R*^2^ = 0.07156) and eosinophil count (*ρ* = −0.6047, *p* = 0.0190; *β* = −15.80, *R*^2^ = 0.3982) negatively correlated with *α-actin* ΔΔCt values. Results are presented in Figure 5. These associations suggest that increased systemic and eosinophilic inflammation is associated with higher *α-actin* and *fibronectin* gene expression in sinonasal tissue.

**Figure 6:**
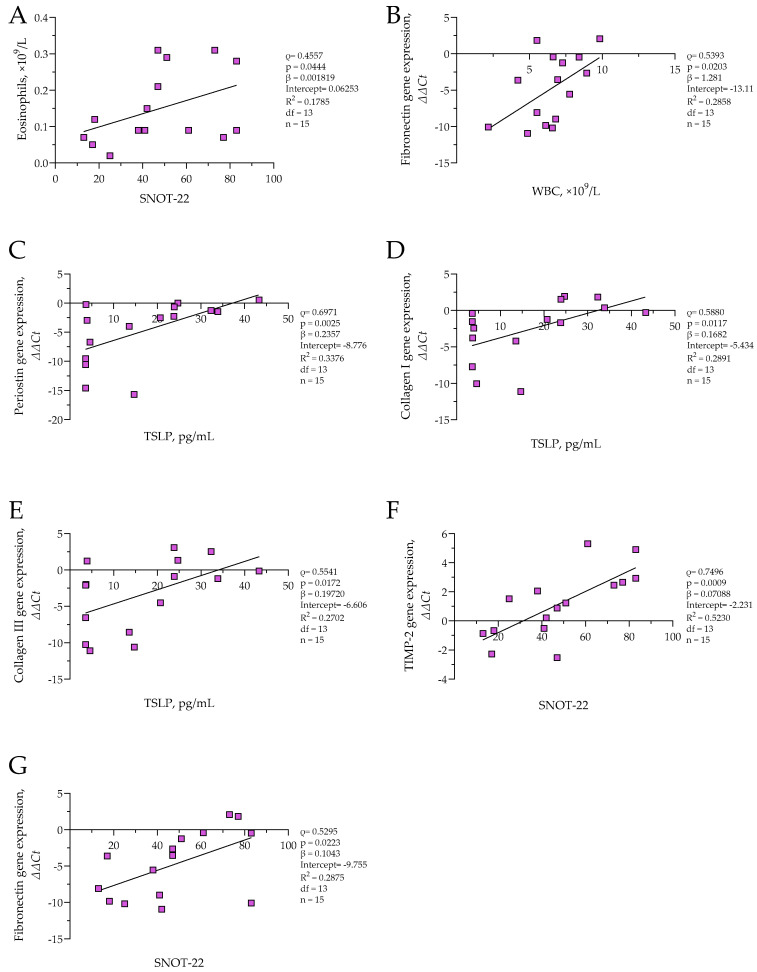
(**A**) Correlation between eosinophil count and SNOT-22 score in CRSwNP group; (**B**) correlation between *fibronectin* gene expression and WBC count in CRSwNP group; (**C**) correlation between *periostin* gene expression and TSLP level in CRSwNP group; (**D**) correlation between *collagen I* gene expression and TSLP level in CRSwNP group; (**E**) correlation between *collagen III* gene expression and TSLP level in CRSwNP group; (**F**) correlation between *TIMP-2* gene expression and SNOT-22 score in CRSwNP group; (**G**) correlation between *fibronectin* gene expression and SNOT-22 score in CRSwNP group. SNOT-22—sinonasal outcome test; TIMP-2—tissue inhibitor of metalloproteinases 2; TSLP thymic stromal lymphopoietin; WBC—white blood cells. Gene expression data are presented as ΔΔCt, with lower ΔΔCt values indicating higher gene expression. CRSwNP n = 15. Statistical analysis—Spearman r test and nonlinear regression.

In the CRSwNP group, eosinophil count was positively correlated with SNOT-22 scores (*ρ* = 0.5357, *p* = 0.0444; *β* = 0.001819; *R*^2^ = 0.1785), and significant positive associations were also observed between symptom SNOT-22 and *TIMP-2* (*ρ* = 0.7496, *p* = 0.0009; *β* = 0.07088; *R*^2^ = 0.5230) as well as SNOT-22 and *fibronectin* (*ρ* = 0.5925, *p* = 0.0223; *β* = 0.1043; *R*^2^ = 0.2875) ΔΔCt values, indicating decreased expression of these remodeling genes in patients with more severe symptoms. A negative correlation was detected between WBC count and *fibronectin* ΔΔCt (*ρ* = 0.5393, *p* = 0.0203; *β* = 1.218, *R*^2^ = 0.2858), suggesting increased *fibronectin* expression with elevated systemic inflammation. Additionally, TSLP protein concentration was positively associated with ΔΔCt values of *collagen I* (*ρ* = 0.5880, *p* = 0.0117; *β* = 0.1682; *R*^2^ = 0.2891), *collagen III* (*ρ* = 0.5541, *p* = 0.0172; *β* = 0.19720; *R*^2^ = 0.2702), and *periostin* (*ρ* = 0.6971, *p* = 0.0025; *β* = 0.2357; *R*^2^ = 0.3376), indicating that higher TSLP levels were associated with reduced expression of these genes. The results are presented in Figure 6. No significant correlations were found in the control group. 

## 4. Discussion

CRS is a chronic inflammatory disease of the mucous membrane of the upper respiratory tract. CRS is one of the most common otorhinolaryngological diseases and inflammatory conditions characterized by chronic inflammation of the mucous membrane of the paranasal sinuses, lasting more than 12 consecutive weeks [24]. A diagnosis based on the symptoms of this disease may be inaccurate since approximately 40% of patients diagnosed with the disease, according to the symptoms, do not develop characteristic endoscopic or radiological signs of the disease [24]. CRS is one of the most common chronic diseases. The symptomatic prevalence is 7% to 27% of adults in European countries, approximately 14% in the USA, and 2.1% to 8% in China [25,26,27]. Chronic sinusitis is a multifactorial disease that includes infectious, inflammatory, or structural factors. Thus, allergic rhinitis, exposures, structural causes, ciliary dysfunction, immunodeficiencies, and fungal infections should be considered [28]. CRS is classified into two phenotypes, CRSwNP and CRSsNP, and into three main endotypes, which are distinguished based on models of T-helpers producing cytokines [29,30]. Several pathogenic factors are believed to be associated with the emergence and development of CRS, primarily with epithelial barrier dysfunction, impaired mucociliary clearance, impaired immune response, and excessive tissue remodeling [31].

The pathogenesis of CRS is primarily linked to the dysfunction of the epithelial barrier, where the epithelium is structurally and functionally abnormal, which can significantly influence the development and progression of this disease [31]. Epithelial cells actively produce antimicrobial peptides, cytokines, and chemokines, which activate intraepithelial and subepithelial cells, incorporating them into the tissues of the respiratory tract and thus maintaining the physical, chemical, and immunological barrier [32,33,34]. Activated epithelial cells release a range of cytokines and biologically active substances that enhance inflammation. Cytokines of epithelial origin include TSLP, IL-25, and IL-33, which can stimulate the production of various Th2 cytokines, such as IL-4, IL-5, and IL-13, by activating Th2 cells and type 2 innate lymphoid cells (ILC2), thus further stimulating type 2 inflammatory responses [10]. Our study found that TSLP concentrations in the plasma of the CRS group were higher than in the control group, showing possible epithelial cell activation. Other studies showed that in patients with CRSwNP, the tissues of nasal polyps accumulate more dendritic cells (DC), expressing IL-17Rb, ST2, and TSLPR on their surface, than the tissues of healthy individuals. DC is activated in response to IL-25, IL-33, and TSLP and can initiate Th2 reactions [35].

This research provides an in-depth examination of tissue remodeling patterns in CRS patients, highlighting the significant differences between the CRSwNP and CRSsNP phenotypes. We aimed to identify potential structural features associated with different phenotypes by combining clinical symptom data, histological findings, gene expression profiling, and plasma cytokine measurements, ultimately to inform more targeted treatment strategies.

Our cohort included 30 CRS patients (15 with CRSwNP, 15 with CRSsNP) and 10 non-CRS controls. Symptom burden measured by SNOT-22 was significantly higher in both CRS groups, showing no meaningful difference between CRSwNP and CRSsNP. In contrast, systemic immune parameters varied: eosinophil counts were markedly higher in CRSwNP, while basophils were notably elevated in CRSsNP. The increased eosinophils in CRSwNP align with previous studies indicating a predominant type 2 inflammation in this phenotype, whereas the raised basophils and neutrophils in CRSsNP suggest mixed or non-type 2 inflammatory processes [36,37].

Histological analysis showed distinct phenotypic differences. In CRSsNP, hyperplasia and thickened basement membranes were common, whereas CRSwNP exhibited more vascularization and eosinophilic infiltration. These observations align with earlier findings, indicating that CRSwNP is associated with an edematous stroma, eosinophilia, and increased angiogenesis, whereas CRSsNP exhibits a denser, fibrotic tissue structure [10]. Interestingly, our findings are in agreement with Shi et al. [38], who reported increased epithelial hyperplasia and fibrosis in CRSsNP tissue compared to CRSwNP. The thickening of the basal membrane in the CRS due to the secretion of TGF-*β* eosinophils leads to the activation of fibroblasts that start to produce ECM proteins [39].

TGF-*β* is a pleiotropic cytokine with many bodily functions; it regulates cell growth, proliferation, differentiation, and apoptosis. Additionally, TGF-*β* plays a vital role in tissue remodeling associated with chronic rhinosinusitis. It has been shown that eosinophil infiltrates are the primary source of TGF-*β* in nasal polyps. The increased expression of TGF-*β* promotes stromal fibrosis, which is evident in the formation of nasal polyps, primarily due to enhanced production of ECM proteins such as collagen and fibronectin [40]. Our study showed that TGF-*β*1 concentration was higher in CRS groups than in the control group, gene expression was higher in CRS groups than in the control group, and CRSsNP had higher gene expression of *TGF-β1* than in CRSwNP group. TGF-*β* also influences the homeostasis of ECM proteins via changed gene expression of ECM proteins, MMPs, and TIMPs, thus promoting remodeling. Our study also found that gene expression of *MMP-2* and *MMP-9* was higher in CRS groups than in the control group, but CRSsNP also had greater values of *MMP-2* and *MMP-9* gene expression than CRSwNP. Literature mainly reports elevated concentrations of MMP-1 (collagenase), MMP-2, and MMP-9 [40].

TGF-*β*1 has an anti-inflammatory effect, which reduces the synthesis of IgE and the activation of eosinophils. In addition to its anti-inflammatory properties, TGF-*β* enables fibroblasts to proliferate and differentiate into myofibroblasts. Myofibroblasts produce substantial ECM proteins, such as fibronectin and types I and III collagens, essential for tissue remodeling [31]. TGF-*β* is the primary factor linked to tissue remodeling in CRS, primarily due to the synthesis of procollagen—the increased accumulation of procollagen results in subepithelial fibrosis. The primary source of TGF-*β* in nasal polyps is tissue infiltration by eosinophils. Elevated expression of TGF-*β* promotes stromal fibrosis, evident during the formation of nasal polyps, mainly due to increased secretion of collagen and fibronectin in the ECM. The proliferation of fibroblasts in nasal polyps may depend on the concentration of TGF-*β*1 [40].

TGF-*β* plays a crucial role in regulating the repair of epithelial cells during the reconstruction of the lower respiratory tract and can significantly influence the resurgence of chronic rhinosinusitis [41].

TGF-*β*1 enhances the production of MMP-2 and MMP-9 in primary nasal fibroblasts via the NF-kB and Smad2/3 signaling pathways. MMP-2 and MMP-9 degrade types IV and V collagen and elastin in the basement membrane [42,43]. TGF-*β*1 is recognized as a significant factor in the resurgence processes associated with chronic sinus disease and serves as the primary switch for various models of CRS remodeling [20]. Additionally, TGF-*β*1 influences fibrosis by enhancing the deposition of ECM components, such as collagen and fibronectin [44]. Our study found that gene expression of *collagen I*, *collagen III*, *fibronectin*, *vimentin*, *periostin*, and *tenascin C* was higher in both CRS groups compared to the control subjects’ post-operative materials, and it was even greater in CRSsNP than in CRSwNP. The increased expression of *α-actin* in CRS fibroblasts suggests potential activation, leading to elevated gene expression of *α-actin*, among the most essential proteins in muscle cells. Myofibroblasts are characterized by actin filaments known as *α*-actin in the cytoplasm, and they play a critical role in fibroblast migration, contractile forces, and ECM production. Activated fibroblasts secrete significant growth factors and cytokines that disrupt normal ECM homeostasis, promoting angiogenesis and epithelialization. Throughout this process, ECM generation and remodeling occur through a dynamic balance between MMPs and TIMPs secreted by nasal fibroblasts. Furthermore, myofibroblasts express *α-actin*, *collagen I*, *fibronectin*, *MMP-2*, and *MMP-9*, which were also identified in our study of gene expression in post-operative material analysis and serve as key markers for fibroblast differentiation to myofibroblasts [18,43]. Activated fibroblasts produce increased levels of TGF-*β*, which support tissue remodeling, including ECM production, hyperplasia, and the infiltration of inflammatory blood cells. *Periostin* gene expression and immunohistochemistry findings were higher in the CRS group than in controls [14]. The differential expression of periostin and osteopontin significantly correlated with TGF-*β*1 [45]. Other studies found that in the CRSsNP group, TGF-*β*1, TGF-*β* receptor-I, and II and Smad3 protein levels were significantly higher than those in the control group [41].

Gene expression analysis revealed that ECM-related genes, including *collagen I*, *collagen III*, *fibronectin*, *vimentin*, *periostin*, *tenascin C*, and *α-actin*, were significantly upregulated in CRS tissues compared to controls. Notably, CRSsNP exhibited consistently higher expression levels of these genes than CRSwNP, indicating a more advanced or fibrotic remodeling process phenotype. Similar findings were reported by Kato et al. [46], who demonstrated upregulated collagen I and *α*-actin expression in CRSsNP, along with their link to elevated myofibroblast activation levels.

Elastin gene expression was downregulated in both phenotypes, indicating compromised elastic fiber integrity in CRS mucosa [18], consistent with the findings of Cho et al. [47], who noted elastolysis and structural tissue fragility in the nasal mucosa.

We also observed a significant upregulation of *TGF-β1* gene expression and protein concentration in CRSsNP, which was notably higher than the levels found in CRSwNP and controls. This aligns with prior literature identifying TGF-*β*1 as a key inducer of myofibroblast differentiation and ECM deposition, particularly in Th1/Th17-dominant CRS endotypes [43]. Brar et al. [48] further confirmed TGF-*β*1 as a key epigenetically regulated factor in fibrotic remodeling within CRS tissue. Increased *α-actin* gene expression, along with correlations between inflammatory markers (WBC, eosinophils) and the expressions of *α-actin* and *fibronectin* genes, indicates active fibroblast-driven remodeling in CRSsNP.

In contrast, remodeling in CRSwNP appears to be more subdued or even hindered, despite the indication of eosinophilic inflammation. Although TSLP levels were found to be increased in CRSwNP, they exhibited a positive correlation with the ΔΔCt values of collagen I, collagen III, and fibronectin. This suggests that individuals with elevated epithelial-derived cytokine activity have a lower expression of these structural genes [14]. This finding challenges the view of TSLP as a solely pro-fibrotic mediator, suggesting a complex regulatory role in the upper airway that potentially modulates epithelial barrier integrity or favors edema over fibrosis. This is supported by Huang et al. [31], who demonstrated that TSLP can inhibit collagen synthesis through non-canonical pathways in nasal epithelial—fibroblast interactions, and by Al-Alawi et al. [34], who observed reduced ECM stiffness and fibroblast contractility in lung tissue with elevated TSLP.

The integration of immune, clinical, and molecular data revealed phenotype-specific patterns. In CRSsNP, symptom severity correlated with WBC count, while remodeling gene expression was positively associated with systemic inflammatory markers, including eosinophils and basophils. This supports a model in which systemic immune activation may drive local fibrotic remodeling and symptom manifestation, echoing findings that show systemic neutrophilic inflammation contributes to mucosal fibrosis [17,49,50,51].

In CRSwNP, however, eosinophil count was positively associated with SNOT-22 scores, while increased symptom burden correlated with reduced expression of TIMP2 and FN1. These findings suggest that in CRSwNP, impaired ECM maintenance or dysregulated remodeling, rather than fibrosis itself, may be associated with disease severity. Prior research also supports that polypoid CRS involves an imbalance in MMP/TIMP activity, contributing to tissue fragility and polyp formation [14,40]. Other studies likewise noted reduced TIMP-2 expression and enhanced MMP-9 activity in CRSwNP biopsies [52].

From a clinical standpoint, these findings underscore the importance of differentiating CRS phenotypes not only by anatomical features but by molecular remodeling profiles. CRSsNP may benefit from therapies aimed at inhibiting TGF-*β*1 signaling and fibroblast activity to reduce fibrotic tissue accumulation, while CRSwNP may require interventions targeting epithelial cytokines (e.g., TSLP, IL-4, and IL-13), vascular remodeling, or basal cell dysregulation.

One notable limitation of our study is the reliance on phenotypic classification of CRS into CRSwNP and CRSsNP rather than endotyping based on eosinophilic versus non-eosinophilic inflammation. Although inflammatory endotyping provides a more accurate reflection of the underlying immunopathology—particularly in distinguishing type 2 eosinophilic CRS from other variants—this classification requires consistent histopathological data, including eosinophil counts, as well as immunoassays for key cytokines such as IL-4, IL-5, and IL-13. Unfortunately, our study was limited by incomplete histological data across the full cohort, and thus, we could not reliably assign patients to endotype-based subgroups. To maintain methodological consistency with prior tissue remodeling research and ensure statistical power, we adhered to the widely recognized CRSwNP vs. CRSsNP phenotypic classification. We acknowledge that future prospective studies incorporating systematic histological and immunological profiling will be essential for validating our findings in the context of CRS endotypes. Furthermore, in Western Countries, Type 2 inflammation is associated with 85% of CRSwNP, while CRSsNP is mainly considered a Type 1 mediated disease [53,54]. In our study, we found that more than 53 percent of CRSsNP and more than 73 percent of CRSwNP had eosinophils as a finding in the histology of post-operative material. Additionally, eosinophils were found in less than 7 percent of CRSsNP and more than 26 percent of CRSwNP in nasal discharge. However, the treatment strategy is usually selected based on endoscopic findings as histology is usually evaluated after surgery.

## 5. Conclusions

This study revealed significant differences in tissue remodeling and inflammatory signatures between CRSwNP and CRSsNP phenotypes. CRSsNP was characterized by increased gene expression of ECM components including collagen I, collagen III, *fibronectin*, and *α-actin*, along with upregulated TGF-*β*1 levels, indicating a pronounced fibrotic remodeling process. In contrast, CRSwNP displayed higher TSLP concentrations but lower expression of structural ECM genes, suggesting an alternative remodeling mechanism, possibly associated with edema rather than fibrosis. Correlations between systemic inflammatory markers and tissue gene expression further confirmed phenotype-specific remodeling patterns. These findings underscore the need for phenotype-specific treatment strategies, targeting fibrotic remodeling in CRSsNP and epithelial cytokine signaling in CRSwNP, to improve outcomes in CRS patients. Personalized therapeutic approaches based on remodeling profiles could enhance long-term disease control and surgical success.

## Figures and Tables

**Table 1:** Primers that were used for gene expression qRT-PCR analysis. MMP—matrix metalloproteinases; TIMP—tissue inhibitor of metalloproteinases; TGF-*β*1—transforming growth factor *β*1.

*18S*	Forward	5′-CGCCGCTAGAGGTGAAATTC-3′
	Reverse	5′-TTGGCAAATGCTTTCGCTC-3′
*Collagen I*	Forward	5′-TCGAGGAGGAAATTCCAATG-3′
	Reverse	5′-ACACACGTGCACCTCATCAT-3′
*Collagen III*	Forward	5′-TATCGAACACGCAAGGCTGTGAGA-3′
	Reverse	5′-GGCCAACGTCCACACCAAATTCTT-3’
*Fibronectin*	Forward	5′-AGCCAGCAGATCGAGAACAT-3′
	Reverse	5′-TCTTGTCCTTGGGGTTCTTG-3′
*Vimentin*	Forward	5′-GCAAAGATTCCACTTTGCGT-3′
	Reverse	5′-GAAATTGCAGGAGGAGATGC-3′
*Periostin*	Forward	5′-TGCCCTGGTTATATGAGAATGGAAG-3′
	Reverse	5′-GATGCCCAGAGTGCCATAAACA-3′
*Tenascin C*	Forward	5′-GAGACATCTGTGGAAGTGGA-3′
	Reverse	5′-CGTACTCAGTGTCAGGCTTC-3′
*α-actin*	Forward	5′-TGGGTGACGAAGCAC AGAGC-3′
	Reverse	5′-CTTCAGGGGCAACACGAAGC-3′
*Elastin*	Forward	5′-GGCCATTCCTGGTGGAGTTCC-3′
	Reverse	5′-AACTGGCTTAAGAGGTTTGCCTCCA-3′
*MMP-2*	Forward	5′-GGCCCTGTCACTCCTGAGAT-3′
	Reverse	5′-GGCATCCAGGTTATCGGGGA-3′
*MMP-9*	Forward	5′-GGCCTCCAACCACCACCAC-3′
	Reverse	5′-CGCCCAGAGAAGAAGAAAAGC-3′
*TIMP-1*	Forward	5′-AGACCTACACTGTTGGCTGTGAG-3′
	Reverse	5′-GACTGGAAGCCCTTTTCAGAG-3′
*TIMP-2*	Forward	5′-ATGCACATCACCCTCTGTGA-3′
	Reverse	5′-CTCTGTGACCCAGTCCATCC-3′
*TGF-* *β1*	Forward	5′-GTACCTGAACCCGTGTTGCT-3′
	Reverse	5′-GAACCCGTTGATGTCCACTT-3′

**Table 2:** Clinical and demographical data of study subjects.

	CRSsNP	CRSwNP	Control Group
Age, mean ± SD [min–max]	53.27 ± 14.06 [28–78]	53.93 ± 14.90 [20–72]	46.90 ± 24.20 [22–87]
Gender, M/F, n	11/4	7/8	7/3
SIRI score	0.93 ± 0.52	0.93 ± 0.60	0.99 ± 0.50
SNOT-22	45.53 ± 18.10 §	47.73 ± 23.75 §	14.80 ± 11.22
Time of disease, years	7.78 ± 14.23	10.75 ± 11.22	N/A
WBC, ×10^9^	6.573 ± 2.260	6.505 ± 1.927	7.003 ± 2.047
Neutrophils, ×10^9^	3.900 ± 1.715	4.148 ± 1.450	4.479 ± 1.851
Lymphocytes, ×10^9^	2.039 ± 0.891	1.816 ± 0.625	1.981 ± 0.661
Monocytes, × 10^9^	0.448 ± 0.185	0.373 ± 0.120	0.415 ± 0.158
Eosinophils, ×10^9^	0.159 ± 0.114	0.149 ± 0.102	0.097 ± 0.090
Basophils, ×10^9^	0.027 ± 0.015 *	0.018 ± 0.014 §	0.031 ± 0.020
Neutrophils, %	57.93 ± 10.74	62.78 ± 9.20	62.62 ± 11.41
Lymphocytes, %	32.16 ± 8.78	28.86 ± 8.26	29.45 ± 9.53
Monocytes, %	6.96 ± 2.06	5.79 ± 1.13	6.06 ± 1.89
Eosinophils, %	2.473 ± 1.685	2.247 ± 1.212 §	1.400 ± 1.173
Basophils, %	0.473 ± 0.333	0.320 ± 0.251	0.470 ± 0.334
IgE, kIU/L mean ± SD [min–max]	290.20 ± 470.40 [3.00–1649.00]	163.00 ± 246.50 [3.00–1342.00]	50.44 ± 58.33 [3.00–170.60]

CRSsNP—chronic rhinosinusitis without nasal polyposis; CRSwNP—chronic rhinosinusitis with nasal polyposis; IgE—immunoglobulin E; N/A—not applicable; SIRI—systemic inflammation response index; SNOT-22—sinonasal outcome test; WBC—white blood cells. Data presented as the mean ± SD, n, and [min–max] values. * *p* < 0.05 compared to CRSwNP; § *p* < 0.05 compared to the control group. Statistical analysis between investigated groups—two-sided Mann–Whitney U test (independent data).

**Table 3:** Cytology and histology findings.

	CRSsNP n = 15	CRSwNP n = 15	Control n = 10
Cytology findings			
Leukocytes (none/a few/some/many/a lot), %	13.3/6.7/20.0/13.3/46.7	−/13.3/6.7/20.0/60.0	12.5/37.5/−/−/50.0
Neutrophils (none/a few/some/many/a lot), %	13.3/−/33.3/-/53.3	13.3/−/13.3/6.7/66.7	12.5/−/37.5/-/50.0
Eosinophils (none/a few/some/many/a lot), %	93.3/−/6.7/−/−	73.3/−/13.3/−/13.3	87.5/−/12.5/−/−
Lymphocytes (none/found), %	93.3/6.7	100.0/-	100.0/-
Plasma cells (none/ found), %	93.3/6.7	100.0/-	100.0/-
Macrophages (none/a few/some/many/a lot), %	93.3/−/6.7/−/−	100.0/−/−/−/−	100.0/−/−/−/−
Mucus (none/found), %	80.0/20.0	80.0/20.0	50.0/50.0
Bacteria (none/a few/some/many/a lot), %	80.0/20.0	66.7/33.3	50.0/50.0
Histology findings			
Hyperplasia (none/found), %	46.7/53.3	93.3/6.7	ND
Vascularization (none/found), %	66.7/33.3	13.3/86.7	ND
Neutrophils (none/found), %	40.0/60.0	80.0/20.0	ND
Eosinophils (none/found), %	46.7/53.3	26.7/73.3	ND
Eosinophilia (≤50/51–100/101–199/≥200), %	25.0/−/75/−	25.0/37.5/12.5/25.0	ND
Lymphocytes (none/found), %	6.7/93.3	6.7/93.3	ND
Plasmocytes (none/found), %	13.3/86.7	6.7/93.3	ND

Results are expressed in percentages of all cases in the group. CRSsNP n = 15, CRSwNP n = 15, control n = 10. CRSsNP—chronic rhinosinusitis without nasal polyposis; CRSwNP—chronic rhinosinusitis with nasal polyposis; ND—not done.

## Data Availability

This article includes all the data presented in this study.

## Abbreviations

The following abbreviations are used in this manuscript:

CRS	chronic rhinosinusitis
CRSsNP	chronic rhinosinusitis without nasal polyposis
CRSwNP	chronic rhinosinusitis with nasal polyposis
DC	dendritic cells
ECM	extracellular matrix
EGF	epidermal growth factor
ILD	innate lymphoid
MMP	matrix metalloproteinases
PDGF	platelet-derived growth factor
TGF-*β*	transforming growth factor
Th	T-helper cell
TIMP	tissue inhibitor of metalloproteinases
TSLP	thymic stromal lymphopoietin
TSLPR	thymic stromal lymphopoietin receptor
SIRI	systemic inflammation response index
SNOT	sinonasal outcome test
